# Supplementary material for: Injury Pathology in Young Gymnasts: A Retrospective Analysis
Source: Children (Basel). 2023 Feb 4;10(2):303. doi: 10.3390/children10020303 (PMC9955164; doi:10.3390/children10020303)
Supplement: Supplementary file 1 [file children-10-00303-s001.zip › children-2167793-supplementary.pdf]

Supplementary online material (file S1) for 'Injury Pathology in Young Gymnasts: A Retrospective Analysis' Injury questionnaire

Parent/guardian information sheet

My name is Emma Williams, I am a PhD student at Cardiff Metropolitan University researching the injury risk factors in youth gymnasts. I am Supervised by Jason Pedley and Rhodri Lloyd in which both hold a doctorate in the area of youth physical development. Both Jason and Rhodri have publications in the area of youth sports as well as lecture in Strength and Conditioning at Cardiff Metropolitan University. As your child is male/female between 6-18 years of age, you have been approached as it is important that both you and your child understand the aims of this study and what is required of you if you wish to participate.

What will the research consist of?

This questionnaire will ask for information regarding the gymnast's previous injury history within the last 12 months. No personal information will be asked other than their sex and age; all data will remain anonymous.

What are the risks of participating in this study?

There is no risk to participating in this study, the answers are confidential, and anonymity will remain for all participants when writing up results from this study.

What will happen to the data and information collected?

All of the data collected in this study is anonymous. Copies of all of the data will be exported onto a password protected external hard drive. Results from this study may be seen by members of the public, however no participants identity will be revealed as all participants are anonymous and only group averages and trends will be reported; individual participant data will not be reported. As the parent/guardian you will be asked to provide your name and email address as proof an adult was present, your details will not be shared and are confidential. As the answers are in regards to your child their details will not be asked in order to ensure their answers are fully anonymous.

What next?

If your child chooses to take part in this questionnaire it is important that they have adult supervision to ensure they have full understanding of each question. Due to such a range of ages that will take part, the questions have been constructed assuming the child will have help from an adult, however the answers (if possible) should primarily come from the child and any details they cannot remember or do not understand should then be completed with the help of an adult. I cannot give guidance as to what age the child can complete this questionnaire alone as educational capability may vary, therefore it is to your own judgment whether they are capable of this. If you have any questions prior to taking part or after participating, please feel free to contact me via email at: sm22050@cardiffmet.ac.uk

Parent/guardian consent form:

I have read the information form regarding the research and fully understand what it involves. The researcher has answered any questions I had, and I feel fully comfortable and satisfied with their response. I understand I am entitled to ask any further questions at any time throughout the duration of the study. Please tick 'fully agree' or 'fully disagree' for the following statements. If you

feel you disagree, the questionnaire will end as the questionnaire cannot be completed without consent.

	fully agree	disagree
I am happy for my child to volunteer to participate on their own accord, and they are entitled to stop completing the questionnaire at any point if they wish to.	○	○
All information collected during the study will be anonymous and exported to a password protected excel file. Results of the study may be published in the future, however, my Child's anonymity will be maintained at all times. Therefore, I consent to this data being used.	○	○
I understand I can contact the researcher at any point if I have any questions and these contact details cannot be linked to my Child's questionnaire responses as no personal information is provided during the questionnaire.	○	○
I am happy to provide my name and email address as the parent/guardian as proof that an adult was present during this questionnaire. I also understand that my details are confidential and will not be shared with the public.	○	○

Please provide your full name and email address as the parent or guardian to confirm an adult was present.

○ Name ____

○ Email address ____

Check this box by way of a signature to confirm you have read and understood this form.

Gymnast information sheet:

What is a study?
A study is what people do when they want to learn about something new!

Why is this study being done?
This study is being done to find out how many people get hurt doing gymnastics and why they might be getting hurt.

What will happen if I do the study?
If you understand this study and feel happy, all you have to do is write your name below and answer some questions. All of the questions have choices to pick from so you don't have to write anything, you just pick the answer that suits you best!

Do I have to say yes?
Not at all! It is your decision if you want to join in.
Nobody will mind if you do not want to join in, and if you change your mind when answering the questions, you can also stop whenever you want.

Will this study upset me?
No- the study will only ask questions about how you hurt yourself. If you find any questions confusing or you are not sure, you can ask your parent or carer.

What will happen to my answers?
All of your answers are top secret! No one will know they are your answers, if the answers from this study are put in a magazine there will be no way people could know you answered any of the questions.

Who can I ask about this?
If you are confused you can ask any questions you have to your parents (or carer) because they have been given lots of information!

Gymnast consent form:
I have read the information form that explains what I need to do if I want to take part in the study. I understand that I can ask my parent/guardian or contact the researcher if I have any questions

before or after taking part. Please tick the smiley face if you are happy and the sad face if you are not...

	:)	:(
I understand I can stop filling out the survey whenever I want to, and no one will ask me why.	○	○
I have decided to take part because I want to, and nobody has made me.	○	○
The results of the study might be put into a book or magazine in the future, but I understand my name cannot be used and nobody will know who I am.	○	○

Write the letters at the start of your first and last name in the box below. e.g. Spider Man would be: SM

End of Block: Information and consent

Start of Block: Demographics

Q2.1 What is your sex?

○ Boy

○ Girl

○ Other

Q2.2 When is your birthday? e.g. day (25), month (April), year (2004)

☐ day ____

☐ month ____

☐ year ____

Q2.3 Where are you from?

- Europe/UK
- USA/Canada
- Africa
- Asia
- Caribbean
- North America
- South America
- Central America

Q2.4 What type of gymnastics do you take part in? If you take part in more than 1 type, please tick each one.

- ☐ Artistic
- ☐ Rhythmic
- ☐ Acrobatic
- ☐ Aerobic
- ☐ Team
- ☐ Trampolining
- ☐ disability
- ☐ Double mini tramp
- ☐ Tumbling

Q2.5 Do you take part in other sports outside of school and gymnastics?

- ○ yes
- ○ no

Q2.6 Please select what sports you take part in.

- ○ Football
- ○ Rugby
- ○ Hockey
- ○ Swimming
- ○ Athletics
- ○ Dancing
- ○ Cheerleading
- ○ Horse riding
- ○ Lacrosse
- ○ Climbing
- ○ Tennis
- ○ Badminton
- ○ Netball
- ○ Basketball
- ○ Rounders
- ○ Cricket
- ○ Cycling
- ○ Squash
- ○ Table Tennis
- ○ Fencing
- ○ Water sports
- ○ Outdoor adventure sports e.g. rock climbing

○ Golf

○ Baseball

○ American Football

○ Futsal

○ Volleyball

○ other ____

Q2.7 How many hours a week do you take part in gymnastics?

slide to how many hours you take part in a week

	0 3 5 8 11 13 16 19 21 24 27 29 32 35 37 40
hours	

Q2.8 How many years have you taken part in gymnastics?

	1 3 5 7 9 11 12 14 16 18 20
years	

Q2.9 Do you think you have 'specialised' in gymnastics?

'sport specialisation' meaning gymnastics is your main sport that takes up most of your after school time.

○ yes

○ No

Q2.10 Do you do competitions or grading in gymnastics?

- ◯ yes
- ◯ No

Q2.11 What level do you compete at?

- ◯ Local/School
- ◯ Regional
- ◯ National
- ◯ International

Q2.12 Please select the apparatus you practice on.

- ☐ Floor
- ☐ Vault
- ☐ Uneven Bars (girls only)
- ☐ Beam (girls only)
- ☐ High bar (boys only)
- ☐ Pommel Horse (boys only)
- ☐ Parallel Bars (boys only)
- ☐ Rings (boys only)
- ☐ Tumble track
- ☐ Double mini-tramp
- ☐ Trampoline
- ☐ Floor with equipment (Rhythmic)

End of Block: Demographics

Start of Block: injury history

Q3.1 In this section you will be asked if you have had an injury doing gymnastics, if you have you will then be asked some extra questions about that injury.
For this study 'injury' is defined as a time you have hurt yourself doing gymnastics or had a on going pain from gymnastics that meant you could not train as normal. For example, you had to miss out certain skills or apparatus because of the pain or injury you had.

In the last 12 months have you been injured because of doing gymnastics? (This being your worst injury)- there will be an option to report a second injury at the end of this survey.	• Yes<br>• No
How long ago did this injury happen?	• 1-2 months ago<br>• 3-4 months ago<br>• 5-6 months ago<br>• 7-8 months ago<br>• 9-10 months ago<br>• 11-12 months ago
Did this injury happen during...?	• Gymnastics competition<br>• Gymnastics practice/training
How did this injury happen?	• During a dismount landing in gymnastics<br>• Falling off equipment or being hit by equipment<br>• During a gymnastics skill using apparatus e.g. during a bar routine, during a cartwheel<br>• Other
What part of the body was injured?	• Head<br>• Neck<br>• Collar Bone<br>• Ribs<br>• Back<br>• Shoulder<br>• Elbow<br>• Wrist/Hand<br>• Arm<br>• Hip<br>• Knee<br>• Ankle/Foot<br>• Leg-thigh<br>• Leg-shin<br>• Other/unsure
What did the medical professional say your injury was?	• Bone break/fracture<br>• Muscle strain (pulled a muscle)<br>• Ligament sprain (e.g. ankle sprain, elbow sprain)<br>• Dislocation (ends of the bone forced out of their usual positions)

	• Overuse<br>• Tendon tear (small or complete tear of the tendon (e.g. Achilles' tendon rupture is a common injury you may have heard of)<br>• Tendinopathy (inflammation of the tendon, usually from overuse)<br>• Joint bursa (inflammation of fluid filled sack near joints and tendons to reduce friction)<br>• Nerve entrapment (trapped nerve)<br>• Skin lesion (cut or break to the skin)<br>• Growth related injury e.g. Osgood-Schlatter's<br>• Other
Did you receive any treatment following the injury?	• Yes<br>• No
What advice for training were you given?	• Complete rest from gymnastics<br>• Gymnastics training but changed to suit my injury<br>• Told to train through my injury
What length of time was taken out gymnastics?	• 1-3 days (minimal severity)<br>• 4-7 days (mild severity)<br>• 8-28 days (moderate severity)<br>• 29 days + (severe)<br>• Career ending (severe)
